# Clinical Lectures on Diseases of the Urinary Organs

**Published:** 1888-12

**Authors:** 


*Clinical Lectures on Diseases of the Urinary Organs.* By SIR HENRY THOMPSON. Pp. 470. Eighth Edition. London: J. & A. CHURCHILL. 1888.

The critic who peruses the eighth edition of the work of our leading authority on urinary diseases has little more to do than chronicle the event. It is easy to find blemishes: in this case, like the spots in the sun, lost in the general effulgence of the orb, they can be discovered only by looking keenly for them.

Sir Henry loves his subject so well that he seems positively to resent interference, for improvement or otherwise, by any one else. Bigelow merely represents a casual advance on the practice of Sir Henry, who was gradually coming to the practice of lithotrity at one sitting. On p. 204 is the drawing of an apparatus which was invented soon after Bigelow's, and is described as "*original* aspirator of the author." Various operations for prostatic enlargement and prostatic retention have been proposed and practised. Reginald Harrison of Liverpool, and McGill of Leeds, have done some good work in this direction, so far as we provincials are able to judge. Sir Henry mentions the name of neither, and very slightingly speaks of their work. An Indian surgeon, who has had an extraordinary experience and marvellous success in litholapaxy, has broken a lance with our author, and is practically ignored. Something is said of the electric endoscope; it will scarcely be credited that Fenwick's name is not even mentioned.

The editor of this Journal, in writing of Supra-pubic Lithotomy in a work on "Abdominal Surgery," had occasion to point out some errors in the history of the operation which Sir Henry provided for his special work on the subject. These corrections Sir Henry has quietly and without acknowledgment adopted in the work before us, and the hapless author is casually referred to in a footnote as "a recent writer" who "has thought proper to call public attention to my error in saying Pierre Franco of Lausanne, substituting 'Touraine.'" It is perhaps scarcely worth mentioning that this "recent writer" has not once printed the word "Touraine" in the whole of his book. It can scarcely be a printer's error, for we are further told "Franco was borne at Touraine," which is news indeed.

These are undoubtedly small blemishes—spots on the sun, as we have said,—and scarcely interfere with the value of the work as a teaching power. But in these days when knowledge is thrown broadcast, and the authority of words and the weight of popular estimation have had to give way to the authority of facts and the weight of scientific experience, nothing will satisfy us that is not candid, outspoken and faithful exposition of truth. And, if it is not right for a great man to ignore the labours of fellow-workers in his field, it is certainly not wrong for a critic to call attention to his faults in history, in courtesy, and in justice.

